# Remodeling an infarcted heart: novel hybrid treatment with transmyocardial revascularization and stem cell therapy

**DOI:** 10.1186/s40064-016-2355-6

**Published:** 2016-06-16

**Authors:** Jessika Iwanski, Raymond K. Wong, Douglas F. Larson, Alice S. Ferng, Raymond B. Runyan, Steven Goldstein, Zain Khalpey

**Affiliations:** Department of Pharmacology, University of Arizona College of Medicine, Tucson, AZ USA; Department of Surgery, Division of Cardiothoracic Surgery, University of Arizona College of Medicine, P.O. Box 245071, 1501N. Campbell Avenue, Tucson, AZ 85724-5071 USA; Department of Physiological Sciences, University of Arizona College of Medicine, Tucson, AZ USA; Department of Cellular and Molecular Medicine, University of Arizona College of Medicine, Tucson, AZ USA; Research and Development, Cryolife, Inc., Kennesaw, GA USA; Banner University Medical Center, 1501N. Campbell Avenue, Room 4302A, Tucson, AZ 85724 USA; Medical Research Building, 1656 E. Mabel St, Rm 120, Tucson, AZ USA

**Keywords:** Laser therapy, Stem cell therapy, Transmyocardial revascularization, Myocardial infarction, Coronary artery disease, CABG, Angina

## Abstract

Transmyocardial revascularization (TMR) has emerged as an additional therapeutic option for patients suffering from diffuse coronary artery disease (CAD), providing immediate angina relief. Recent studies indicate that the volume of surgical cases being performed with TMR have been steadily rising, utilizing TMR as an adjunctive therapy. Therefore the purpose of this review is to provide an up-to-date appreciation of the current state of TMR and its future developmental directions on CAD treatment. The current potential of this therapy focuses on the implementation of stem cells, in order to create a synergistic angiogenic effect while increasing myocardial repair and regeneration. Although TMR procedures provide increased vascularization within the myocardium, patients suffering from ischemic cardiomyopathy may not benefit from angiogenesis alone. Therefore, the goal of introducing stem cells is to restore the functional state of a failing heart by providing these cells with a favorable microenvironment that will enhance stem cell engraftment.

## Background

A significant number of patients currently suffering from coronary artery disease (CAD) experience severe ischemia due to multi-vessel atherosclerotic obstruction, leading to heart failure and impaired myocardial function (Allen et al. 2008). Prophylaxis and treatment for this patient population involves drug therapy, percutaneous coronary interventions (PCI) and coronary artery bypass grafting (CABG) (Allen et al. 2008; Kim et al. 2002). A large portion of these patients suffer from refractory CAD not amenable to percutaneous or conventional surgical interventions (Andréll et al. 2011; Allen et al. 2008; Kim et al. 2002). For this patient population the extent of CAD is widespread and traditional revascularization alone is not sufficient to reinstate adequate flow through the coronary vessels. Transmyocardial revascularization (TMR) has emerged as an additional therapeutic option. It has been reported to provide symptomatic angina relief with improved quality of life, decreased cardiac events and decreased cardiac re-hospitalizations (Allen et al. 1999; Gowdak et al. 2008; Reyes et al. 2010; Tasse and Arora 2007).

Within the past decade, research has encompassed the use of stem cells in conjunction with TMR as a novel dual therapeutic option (Dallan et al. 2008; Patel et al. 2007; Spiegelstein et al. 2007; Shahzad et al. 2012). Even though TMR procedures may provide increased vascularization within the myocardium, patients suffering from ischemic cardiomyopathy with depressed ventricular function may not benefit from angiogenesis alone. Therefore, with the introduction of stem cells the goal is to restore the functional status of a failing heart by enhancing stem cell homing and engraftment. Due to the regenerative capabilities of stem cells, (Tavris et al. 2012; Samuels et al. 2004; Horvath 2008; Lindsay 2003) it is predicted that concomitant injection with TMR may produce a synergistic effect that will improve cardiac function by decreasing patient angina and aiding in myocardial recovery, repair and regeneration.

## Operative technique

**Fig. 1:**
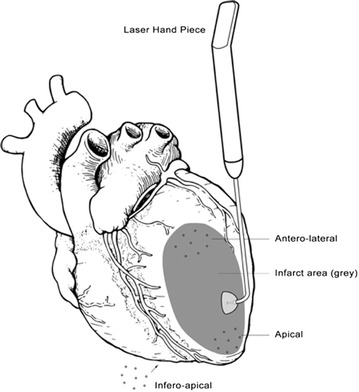
Transmyocardial revascularization (TMR). TMR is performed on the heart by lasing channels in the myocardium, with an energy output of 7 W per laser pulse using the Ho:YAG fiber optic hand tool. The *grey region* seen on the heart represents an infarcted zone following ischemic damage. Depending on the ischemic region and size of the patient heart, a total of 20–40 channels are created using the TMR laser hand piece. Typically, channels are placed on the antero-lateral, apical, and infero-apical regions of the heart

**Fig. 2:**
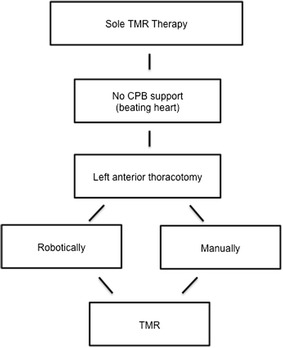
Operative technique for stand-alone TMR therapy. Without CPB support a left anterior thoracotomy can be performed with a robotics device or manually through the fifth intercostal space. TMR is then performed on a beating heart

TMR therapy can be performed with or without adjunctive CABG or other surgical procedures and it can be utilized with or without cardiopulmonary bypass support. For stand-alone laser therapy, a left anterior thoracotomy is performed in the fifth intercostal space, allowing exposure of the pericardium and left ventricular epicardial surface (Fig. 1). Typically 20–40 channels are generated within the left ventricle, depending on the ischemic region and size of the patient heart. Channels are first employed on the inferior surface, moving towards the apex of the heart and subsequently on the lateral and anterior aspects of the epicardial surface (Frazier et al. 2004; Tavris et al. 2012) (Fig. 2).

**Fig. 3:**
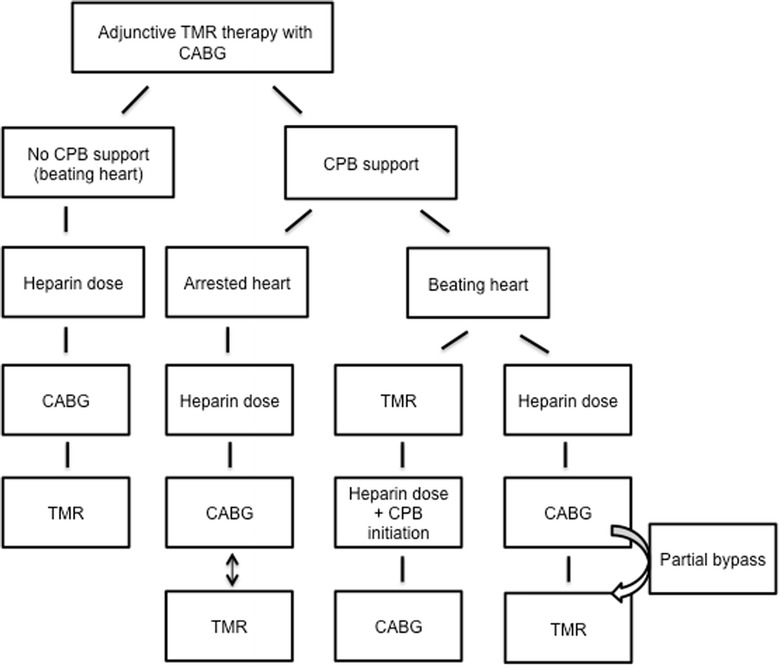
Operative technique for adjunctive TMR therapy. Surgery can be completed with or without CPB support. If CPB is used with an arrested heart, TMR and CABG can be performed according to surgeon preference. However if CPB is utilized with a beating heart, TMR can be performed prior to CPB initiation or it can be performed following bypass grafting on full or partial bypass

Considering most TMR procedures are performed concomitantly with CABG (Tavris et al. 2012), a median sternotomy and CPB support can be utilized during adjunctive therapy (Fig. 3). Under these conditions, TMR channels can be created on an arrested or beating heart depending on the type of laser being employed. The carbon dioxide laser power source is only used on a beating heart with ECG synchronization while the holmium:yttrium–aluminum–garnet (Ho:YAG) TMR laser can be operated on a beating or arrested heart during bypass (Samuels et al. 2004; Horvath 2008). If the surgeon performs the procedure on a beating heart after CPB, laser therapy can be completed following bypass grafting in order to create channels in areas that are not bypassable via grafts or that may provide inadequate blood supply. However if laser therapy is performed before bypass grafting and CPB (Frazier et al. 2004), initially bleeding can be controlled and will subside by the time surgery is complete (Samuels et al. 2004). In addition, TMR therapy can be employed after the CABG procedure, while remaining on bypass with a beating heart (Ak et al. 2009). In this setup the patient can be placed on partial CPB, allowing for increased left ventricular filling and better tactile and auditory responses during channel creation. Lastly, if CPB support is used CABG and TMR can be performed in either order on a still heart, avoiding ventricular arrhythmias and controlling excessive channel bleeding (Samuels et al. 2004).

## Types of laser devices

There are two main types of TMR lasers that are currently approved for surgical use, the carbon dioxide (CO_2_) and the holmium:yttrium–aluminum–garnet (Ho:YAG) laser system (Allen et al. 2008; Horvath 2008). Both create 1 mm wide channels within the myocardium that traverse the ventricular wall (Deckelbaum 1994). The CO_2_ laser system uses carbon dioxide gas excitation to generate infrared light and ablate surrounding tissue. Since the CO_2_ laser can only be delivered to a beating heart, the laser should be pulsed when the heart is quiescent or when it is electrically minimally active, to reduce the risk of arrhythmias (Samuels et al. 2004). This state corresponds to the time lapse between the R and T wave of an ECG rhythm. By avoiding laser pulsation during myocardial contraction, the CO_2_ laser prevents interruption of electrical signaling within the heart, increases laser accuracy and prevents laser firing to non-targeted areas of the myocardium, ultimately reducing the risk of fibrillation (Rudko and Linhares 1992). Once the laser is deployed in a single pulse, the lased energy is transmitted through the myocardial tissue and dissipated within the ventricular blood, preventing excessive thermal injury to surrounding tissue. The CO_2_ laser can deliver 800 W in one pulse lasting from 1 to 99 ms at energies of 8–80 J (Allen et al. 2008).

In contrast, the Ho:YAG laser system can deliver 7 W per pulse with five pulses being delivered per second to a beating or non-beating heart (Allen et al. 2008; Horvath 2008). Unlike the CO_2_ laser, the Ho:YAG laser delivers solid-state holmium:YAG through a fiber optic bundle with pulsation. Channels are placed one centimeter apart and are pulsed on the anterior, lateral, and inferior walls of the left ventricle (Gowdak et al. 2008). Typically 10–20 pulses are necessary to achieve successful penetration via tactile and auditory feedback (Samuels et al. 2004; Horvath 2008).

## TMR mechanisms of action and kinetics

There are 3 competing mechanisms considered to be the source of improved myocardial oxygenation, angina relief and cardiovascular function. The first mechanism focuses on the mechanical perturbations or channels that are created within the myocardium. Termed the patent channel theory, historically this model postulates that TMR creates open conduits that provide the surrounding ischemic myocardium with nutrients and oxygen, acting as sinuses that have the potential to communicate with neighboring cardiac capillary beds. However, this theory has been received with much controversy, as studies show variable channel patency at various time intervals. Hardy et al. (1987) reported channel patency in female puppies for 2 weeks using the CO_2_ laser as compared to a 48 h patency time using a simple needle puncture as a control. Alternatively channel patency has been observed in humans for at least 3 months post mortem via histological measures (Cooley et al. 1994). Although this theory received support early on in TMR development, it has currently been rejected. Acutely, blood flow through lased channels stops momentarily and histology stains display early postoperative clot formation (Mueller et al. 1999).

A model focusing on the denervation of the myocardium has attained increased acceptance as a probable explanation for the short term effects noted after TMR therapy and for the decline in angina score following laser intervention. TMR is thought to disrupt the sympathetic afferent nerve fibers within the myocardium, therefore interrupting pain signaling (Allen et al. 2008). Sympathetic denervation was observed in 6 out of 8 TMR patients with an average increase in sympathetic denervation of 27.5 ± 15.9 % (p = 0.03) within 2 months of treatment (Al-Sheikh et al. 1999). The denervation hypothesis appears to be responsible for the acute effects of TMR, resulting in pain cessation and angina relief almost immediately following surgery. Cardiac nociceptors and afferent fibers are located on the superficial layer of the myocardium, allowing for communication with the brain. Since these receptors are located topically they are easily accessible by the TMR laser. Further examination of the denervation theory suggests that efferent fibers may be denervated following laser therapy. As the active sites of norepinephrine production, denervating these areas would provide β-blockade and thus reduced inotropic support, leading to decreased oxygen consumption and angina relief (Cardarelli 2006).

**Fig. 4:**
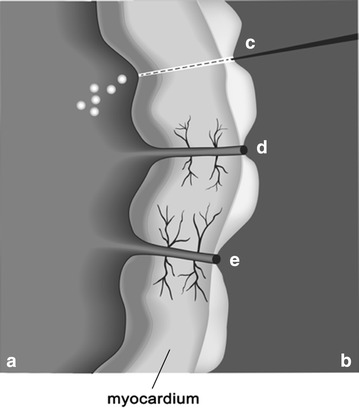
Promotion of angiogenesis. A cross section of myocardium during channel creation with a TMR laser is shown and the interface between the inner (*a*) and outer (*b*) surface of the heart is depicted. The path of channel formation with the laser is depicted in *c*, with steam bubbles arising on the blood side due to heat generation during lasing. Clot formation and angiogenesis are shown at the site of the channel (*d*) and *e* as angiogenesis continues to be stimulated over time

Since denervation provides a plausible explanation for short-term angina relief, a secondary mechanism must be accountable for the long-term effects associated with TMR. Many studies have reported increased vascular density post-TMR treatment suggesting that this laser therapy can induce angiogenesis (Fig. 4). If so, such a theory could explain the increase in myocardial perfusion that is observed following laser therapy, especially since the patent channel theory is currently being debated. Histological samples taken from deceased patients indicated the presence of necrotic tissue and inflammation at days 3 and 16. However, at day 150 fibrous scarring and increased capillary networks were seen (Gassler et al. 1997). Interestingly, this data demonstrated that injury within the myocardium can increase blood flow and that a certain degree of thermal injury is required to induce this flow. This implies that vascular growth is mediated by an inflammatory response in lased regions of the myocardium. Furthermore, the response is specific to thermal-induced laser energy and/or wavelengths, as increased myocardial flow and neovascularization has consistently been observed in laser systems that employ tissue injury as compared to those that do not, such as sham or excimer lasers (Hughes et al. 2000). Therefore, it is the inflammatory-vasculogenic mechanisms that are responsible for the increased vascular network that accompanies TMR therapy.

## In vivo stand alone therapy

Individuals who have a history of multiple percutaneous or surgical procedures and who present with severe diffuse CAD fall into the category of stand-alone therapy and do not meet the criteria for further PCI’s or surgical interventions. The benefits of this therapeutic option have shown promising results over conventional medical management treatments. A study completed in 1999 followed 275 patients with class IV refractory angina for 2 years (Allen et al. 1999). These patients were not eligible for PCIs or CABG procedures and were thus partitioned into a group receiving TMR therapy followed by medical management or medical therapy alone. Although survival estimates for both groups were similar, those that received TMR had an increased improvement in angina relief, higher freedom from cardiac events (i.e. arrhythmia, myocardial infraction, or congestive heart failure), decreased re-hospitalizations due to cardiac events, increased quality of life scores and increased exercise tolerance stress tests (Allen et al. 1999).

The positive results found in this study prompted other retrospective and prospective studies to be completed. Collectively, these studies have examined various endpoints including operative mortality, long-term survival, angina relief, quality of life and exercise testing. After following individuals for 5 years, Allen et al. (2004) observed a decrease of two or more angina classes in 88 % of TMR patients as opposed to 44 % in medically managed patients (p < 0.001). In addition, the average mortality rate after 1 year was determined as 8 % for TMR patients and 13 % for medically managed patients (p = 0.03). Consequently, the observed decrease in angina relief appears to be sustained beyond a single year with promising mortality rates in those treated with laser therapy.

Despite the convincing results found in decreased angina and mortality, the debate over whether TMR therapy augments LV function, in particular LVEF and myocardial perfusion, continues to ensue with inconsistent evidence. While some groups report enhanced perfusion and hemodynamic function (via blood flow and cardiac output respectively) following laser therapy (Atluri et al. 2008) others have disregarded this notion. Tasse and Arora (2007) summarized two studies where there were no significant differences in perfusion between TMR versus medical therapy groups following SPECT or dypiridamole-thallium stress testing. Conversely, they also reported an alternate study which observed a decrease in ischemia at 3, 6, and 12 months in TMR patients, compared to an increase in ischemia in patients receiving medical treatment alone, at the same time intervals (Tasse and Arora 2007). Further comparison of LVEF from different studies by the same authors indicated no significant change after 1 year in LVEF for TMR treated groups as compared to controls in one study and a significant decrease in LVEF by 3 % in another TMR group versus control study (Tasse and Arora 2007). Despite the conflicting data surrounding ventricular function, clear evidence supports the finding that patients receiving TMR typically require decreased drug therapy, in particular short acting nitrates, whereby the number of sublingual nitrates taken per month was reduced from 22.1 ± 30.4 to 1.4 ± 3.9; p < 0.001 (Reyes et al. 2010; Dallan et al. 2008; Gowdak et al. 2005). Decreased cardiac hospitalizations were noted at 12 months following laser treatment, averaging 61 % versus 31 % (p < 0.001), compared to medically managed patients (Tasse and Arora 2007).

## TMR therapy as an adjunct to surgical procedures

Another option for patients with diffuse CAD is to undergo a CABG procedure in order to obtain partial revascularization, and to supplement non-vascularized regions with TMR. 1 year survival rates have been reportedly higher for CABG and TMR therapies as opposed to CABG alone (95 versus 89 %, p = 0.05) (Allen et al. 2000). In addition, there have been no increased operative mortality rates associated with adjunctive therapy compared to sole CABG treatment (1.5 versus 7.6 % respectively, p = 0.02) (Allen et al. 2000) and there have been significant decreases reported in 30 days mortality rates (13 versus 28 %, p = 0.021) (Frazier et al. 2004). More importantly, studies have demonstrated that using TMR in combination with CABG has led to decreased repeat revascularizations (0 dual therapy versus 24 % CABG alone, p < 0.05) (Frazier et al. 2004) and significantly reduced inotropic support post-operatively (Allen et al. 2000). From these studies it has become evident that CABG plus TMR therapy is beneficial over sole CABG treatment since it provides increased angina relief and appears to reduce repeat revascularizations, as well as associated cardiac adverse events.

The question of whether the type of laser used is of importance in these procedures and whether surgical centers are choosing to include laser therapy within their scope of practice, has been evaluated by Tavris et al. (2012). They investigated 435 cardiothoracic hospitals within the United States over the course of 6 years, examining 15,386 patients. They categorized individuals into two procedure groups: those receiving TMR therapy alone, and those receiving TMR with CABG. They also subdivided these groups according to the type of laser (either CO_2_ or Ho:YAG) utilized to determine if there are any significant short or long term effects between laser devices. For TMR therapy performed in isolation or in combination with CABG, both lasers exhibited negligible long-term (>5 years) differences in morbidity and mortality rates. However, short-term results indicated minor odds of morbidity, mortality, and operative mortality with the CO_2_ laser, owing to increased ventilation times. Additionally, short term results for adjunctive therapy reported increased incidences of acute renal failure, deep sternal wound infection, and operative mortality rates for the Ho:YAG laser, but decreased reoperation rates due to bleeding complications (Tavris et al. 2012).

Overall, this data demonstrates that there were no differences in the long-term effects between the two devices, however the Ho:YAG laser appeared to have increases in short-term consequences following dual therapy. It has been previously reported that the Ho:YAG laser introduces increased thermoacoustics (Fisher et al. 1997), thermal injury (Kitade et al. 1999) and tissue tearing (Genyk et al. 2000) to the surrounding muscle. However, it has also been argued that despite increased myocardial damage, it is this damage itself that is paradoxically responsible for the repair and remodeling of ischemic myocardium. Despite these observed repair mechanisms, Estvold and colleagues argued that increased fibrosis associated with the Ho:YAG device led to decreased improvements in ventricular wall thickening and function compared to the CO_2_ laser system (Estvold et al. 2010). Therefore, debate still exists over the extent of injury incurred by these laser devices and their subsequent effects on cardiac function.

## Stem cell treatment with TMR therapy in an infarcted myocardium

The use of stem cells to treat damaged, ischemic zones within an infarcted myocardium has been explored extensively. Nascent cardiac stem cells residing in the myocardium are in limited supply, short lived and rely on immunosuppression—therefore, they can only offer minimal intrinsic regenerative capabilities for patients (Chou et al. 2014). Even though TMR is thought to induce signaling via myocardial injury and inflammation, the quantity and duration of native stem cell homing has been heavily debated. The use of injected stem cells is thought to overcome this hurdle and aid in cardiac repair post-myocardial infarction. Mesenchymal stem cell therapy as a treatment for myocardial pathology is of particular interest due to its regenerative properties aiding in heart failure treatment. The idea that such stem cells or partially differentiated stem cells can be delivered into hearts to restore function is an appealing notion since these cells possess high yields, are multipotent and play a critical role in myocardial repair (Hare et al. 2009; Dinsmore and Dib 2008; Chou et al. 2014; Nagaya et al. 2004). They have displayed significant contributions in myocardial recovery through mechanisms such as transdifferentiation, paracrine release, stimulation of native cardiac stem cells, and inflammatory control (Chou et al. 2014). Although the current paradigm for stem cell therapy focuses on their potential to differentiate into cardiomyocytes and promote cardiac repair and regeneration (Rangappa et al. 2003; Madonna et al. 2009), it has been difficult to demonstrate persistence and biodistribution of stem cells (Spiegelstein et al. 2007). Injection of these cells into the myocardium has exhibited suboptimal retention rates, reportedly ranging from 2.6 to 11.3 % (Hou et al. 2005; Dib et al. 2011). The quick clearance of injected stem cells has been deemed a consequence of myocardial contraction, lymphatic and venous drainage (Richardson et al. 2013) and the hostile nature of an ischemic myocardium (Richardson et al. 2013; Reinecke et al. 1999; Robey et al. 2008; Abdelwahid et al. 2015). This limiting factor in myocardial repair is of significance since successful cell therapy is dependent upon cell homing, engraftment, and survival within the scarred myocardial tissue (Horvath et al. 2005; Richardson et al. 2013). The use of TMR with stem cell therapy could provide a promising solution to this problem and aid in stem cell homing to injured sites.

It is proposed that the inflammatory response imposed by lased thermal injury may be responsible for increased cell homing and engraftment (Shahzad et al. 2012; Patel et al. 2007) as well as angiogenesis (Hughes et al. 2000). Preliminary porcine and murine research has indicated that pretreatment of an infarcted myocardium with TMR therapy significantly enhances mesenchymal stem cell (MSC) engraftment (Patel et al. 2007; Spiegelstein et al. 2007). This engraftment can further be heightened by MSC transfection with vascular endothelial growth factors (VEGF), basic fibroblast growth factors (bFGF), and insulin-like growth factors (70 ± 26 % survival of transfected cells with TMR compared to 47 ± 18 % survival of non-transfected BMSC with TMR, p < 0.05). Transfected cells with laser therapy also experience an increase in vascular density as compared to TMR alone as early as 3 days following injections (Spiegelstein et al. 2007). The success of growth factor mediated repair was further explored by the use of recombinant human basic fibroblastic growth factors (rhFGF-2) and adenoviral fibroblast growth factors-2 (AdFGF-2) with remarkable results in an infarcted porcine model (Horvath et al. 2005; Lutter et al. 2002). Both demonstrated increased contractility and angiogenesis, with an 89 % contractility improvement in the AdFGF-2+TMR group compared to either AdFGF-2 or TMR alone, p = 0.001 via cine MRI (Horvath et al. 2005). Thus, dual growth factor and laser therapy may prove to be effective in candidates with ischemic myocardium undergoing CABG or TMR therapy.

The mechanism(s) underlying the reparative effects of TMR and cell therapy are still heavily debated. It could be that increased perfusion via channel conduits increases cell implantation, prior to eventual channel closure, thus allowing for improved engraftment and angiogenesis. Furthermore the release of pro-survival cytokines (Spiegelstein et al. 2007) or pro-angiogenic growth factors such as FGF-2 (Patel et al. 2007), from inflamed zones may warrant enhanced cell transplantation survival. Current findings also suggest that injection of MSC may mobilize endothelial progenitor cells (EPC) to infarcted zones, aiding in the induction of angiogenesis and the reparative effect noted with TMR (Shahzad et al. 2012). Atluri et al. (2008) demonstrated that sole TMR therapy in a porcine model was able to increase the amount of circulating EPCs post treatment due to the upregulation of NFkB (42.6 ± 27 intensity units versus 591.6 ± 383 intensity units, p = 0.03). NFkB is a potent chemokine and mediator in vasculogenesis, known to excite various vasculogenic agents (VEGF and bFGF). It is thought that it may be responsible for the increase in EPCs within the infarcted myocardium, aiding in vasculogenesis and myocardial perfusion. Moreover, TMR therapy has been shown to upregulate angiopoitein-1 expression (0 ± 0 intensity units vs. 241 ± 87 intensity units, p = 0.003), which has been strongly associated with vasculogenesis and the maturation of vessels (Atluri et al. 2008). Therefore, together with stem cell injection, it is quite evident that both laser and cell therapies can offer synergistic angiogenic effects within scarred, infarcted myocardium.

Clinical assessments following autologous intramyocardial injection of MSC and TMR therapy have yielded promising results for patients with limited therapeutic options. Reports of decreased angina classification of at least two functional classes (from 3.7 ± 0.2 to 1.3 ± 0.2, p < 0.0001) (Dallan et al. 2008) and improved quality of life have been unanimous amongst all case reports (Reyes et al. 2010; Gowdak et al. 2008; Dallan et al. 2008; Konstanty-Kalandyk et al. 2013). Evaluation of ischemic score and LV thickening via MRI have also demonstrated significant improvements 6 months post treatment, with an average decrease in ischemic score from 1.56 ± 0.09 to 0.93 ± 0.10 (p = 0.01) (Gowdak et al. 2008; Dallan et al. 2008; Gowdak et al. 2005). Ischemic scores, measured with MRI during pharmacological stress with dypiridamole, have been used as determinants of myocardial perfusion, while LV thickening has been utilized as a predictor of contractility following therapy. Konstanty-Kalandyk et al. (2013) further examined these parameters concluding that LV segments treated with bone marrow derived MSC + TMR (BMLR) have increased thickening compared to baseline (53.0 ± 7.5 vs. 45.0 ± 9.5 %; p = 0.06) at 1 year post-treatment (Konstanty-Kalandyk et al. 2013). More importantly, they reported that in all but one patient, LV regions treated with BMLR did not demonstrate new infarctions, whereas regions left unattended by BMLR revealed new or enhanced infarcted areas. Ejection fractions, measured pre- and post-treatment, demonstrated conflicting results, concurrent with numerous other study findings. Although Gowdak et al. reported an LVEF improvement from 27 to 43 % in their case report, other institutions have dismissed the idea that TMR and/or cell therapy has an effect on LVEF (Allen et al. 1999; Patel et al. 2007; Klein et al. 2004; Gowdak et al. 2005).

However more recent outcomes in clinical trials have yielded interesting results for sole stem cell therapy. In a meta-analysis published by Kandala et al. (2013), the effect of bone-marrow derived stem cells on ischemic cardiomyopathy was analyzed. By examining over 519 patients and 10 clinical trials they determined that LVEF increased by 4.48 % (p = 0.0001) within 6 months. Furthermore they reported superior effects on LVEF with intramyocardial injections as opposed to intracoronary injections (5.13 %, p < 0.0001 versus 2.32 %, p = 0.3 respectively). Other study findings included significant reductions in LV end-diastolic and end-systolic volumes within stem cell treated patients (Kandala et al. 2013). These results were further supported in a larger meta-analysis examining over 31 randomized controlled trials and 1521 patients with ischemic and non-ischemic cardiomyopathy (Fisher et al. 2015). Administration of any autologous cell therapy via any route was included in the study criteria. Primary endpoints revealed a significant reduction in mortality ≥12 months with patients who received cell therapy alone (p < 0.0001) as well as significant decreases in re-hospitalization rates among stem cell patients with heart failure (p = 0.002). No significance was noted between control and treatments groups in regards to long-term follow-up of arrhythmias (p = 0.61), however significant changes were seen in long-term follow-up of LVEF. Mean increase for LVEF was reported as 4.02 % (p = 0.007) and 3.57 % (p = 0.03) for ischemic heart failure patients alone. Secondary outcomes displayed significant improvements in exercise tolerance (p = 0.02) and quality of life (p = 0.0003). Myocardial perfusion, however, showed conflicting results. Four trials reported significant increases in perfusion while three trials found no significance. These findings can suggest two points. One, that stem cell therapy alone may be sufficient enough to provide therapeutic effects in patients suffering from heart failure. It may be that there short-term engraftment can suffice in providing patients with long-term benefits. Two, that TMR may augment the short-comings of stem cell therapy and provide increased longevity of stem cell engraftment and therefore increased therapeutic effects. An example of this would include studies which report increased myocardial perfusion following TMR (March 1999), and studies which showcase a decrease of two or more angina classifications post TMR (Leon et al. 2005).

## Cost analysis

The decrease in cardiac hospitalizations associated with TMR has not only benefited patient outcomes but it has also been associated with significant hospital cost reductions. The average cost for a patient admitted for angina is estimated to be $3000 per visit. Clinical trials indicate that TMR can reduce these visits by as much as 80 %. Campbell et al. (2001) analyzed 188 patients receiving TMR therapy or medical management at a single UK centre. The total cost for TMR therapy was quoted as $16,500 per patient, with $3480 of that cost coming from CO_2_ laser equipment use and $8630 from inpatient resource use. Alternatively the cost for medical management alone was $3720 per patient with $2900 coming from inpatient and outpatient episodes (Campbell et al. 2001). Although this paper concluded that TMR plus medical management is not cost effective from a UK National Health Service perspective, the periodical *Cost Management in Cardiac Care* (*1998*) argued that TMR has decreased costs of hospitalization time and transfusion rates. Furthermore the average cost of bypass surgery is $40,000 and an angioplasty with a stent can amount to $20,000 with possible need for repetition. On the other hand TMR alone costs an average of $25,000 with quicker recoveries and shorter hospital stays as compared to complete bypass surgery. Although TMR may reduce expenses in the long run versus medical management, this cost-benefit analysis must be closely considered when performing adjunctive CABG procedures and stem cell therapies. A more recent multi-centre study analyzed the cost of mobilizing, harvesting and cryopreserving autologous blood progenitor cells (Mishra et al. 2005). Although the intent of use for these cells was to treat malignant lymphomas and multiple myeloma’s, the study highlights expenses associated with autologous cell delivery. The mean cost of processing stem cells amounted to $6544 per patient. The majority of this expenditure came from hospitalization, growth factors and cryopreservation. Therefore patients considering stem cell therapy should be aware of additional costs associated with this treatment.

## Future research

Laser therapy was first employed for use with patients who could not be revascularized, offering angiogenic properties to ischemic myocardium. Currently, this therapy has been extended to treat scarred, ischemic myocardium with the addition of cell therapy. It is this hybrid treatment that can offer reparative and regenerative properties to injured myocardial tissue in addition to angiogenesis or denervation alone. Therefore, the direction of future research depends on the ability to increase cell homing and engraftment of these cells in order to allow proper repair and function of diseased hearts. Thus far, the literature has reported the use of MSC with laser therapy and their beneficial effects. Due to positive feedback from this dual therapy within the clinical setting it is now important to delineate the precise mechanisms responsible for the aforementioned improvements in cardiac function and determine whether these effects can sustain long-term benefits for patients. Clearly, an alteration in the ischemic microenvironment of the myocardium via laser energy is allowing for increased cell engraftment and survival. Therefore, the attempt to understand the clinical reverberations of this hybrid therapy should focus on the molecular and metabolic interactions occurring at the cellular level.

## Conclusion

TMR has emerged as a promising therapeutic option for patients suffering with diffuse coronary artery disease. The use of TMR within this subgroup has demonstrated many beneficial effects, not only in the quality of life but also in the repair and remodeling of myocardial tissue. To date, this therapeutic option has exhibited encouraging improvements within an ischemic heart model, with angiogenesis acting as the main contributor to the long-term effects noted in patients. Considering that the average amount of tissue affected by this laser treatment is 2 g of muscle (using the Ho:YAG device with 40 lased channels), which corresponds to approximately 1.7–3.2 % of left ventricular mass (Dallan et al. 2008), it is remarkable how this therapy can produce significant angina relief and angiogenesis. The use of cell therapy in conjunction with TMR heightens this response and provides another avenue by which an ischemic myocardium can be revascularized. The future of TMR therapy and research hinges on its collaboration with cell therapies in order to further enhance myocardial repair, regeneration and revascularization.

## Abbreviations

CAD: coronary artery disease
TMR: transmyocardial revascularization
CABG: coronary artery bypass grafting
PCI: percutaneous coronary interventions
CPB: cardiopulmonary bypass
HF: heart failure
